# Letter to the editor: Testing on external independent datasets is necessary to corroborate machine learning model improvement

**DOI:** 10.1093/bioinformatics/btad327

**Published:** 2023-05-18

**Authors:** Giulia Ilaria Corsi, Christian Anthon, Jan Gorodkin

**Affiliations:** Department of Veterinary and Animal Sciences, Center for non-coding RNA in Technology and Health, University of Copenhagen, 1870 Frederiksberg, Denmark; Department of Veterinary and Animal Sciences, Center for non-coding RNA in Technology and Health, University of Copenhagen, 1870 Frederiksberg, Denmark; Department of Veterinary and Animal Sciences, Center for non-coding RNA in Technology and Health, University of Copenhagen, 1870 Frederiksberg, Denmark

The genome engineering revolution of the past decade has been fueled by the development of several computational tools for the design of experiments mediated by the CRISPR/Cas9 endonuclease (Dimauro *et al.* 2022). A crucial step in the design phase is the selection of a guide RNA (gRNA) matching a specific target on the DNA to be cleaved. The target must be upstream of a short sequence motif, named PAM (Jinek *et al.* 2012). To predict the editing efficiency for a gRNA, machine and deep learning models have been trained on datasets of cleavage efficiency measured as indel frequency at endogenous or surrogate targets, or as loss of function readouts of the proteins encoded by the targeted genes (Doench *et al.* 2014, Chari *et al.* 2015, Xu *et al.* 2015, Doench *et al.* 2016, Haeussler *et al.* 2016, Kim *et al.* 2019, Wang *et al.* 2019, Xiang *et al.* 2021, Anthon *et al.* 2022). Measuring cleavage efficiency at surrogate targets by high-throughput sequencing allows efficient production of datasets much larger than those obtained from endogenous cleavage or functional knockouts. Hence, deep learning models for gRNA efficiency prediction use surrogate-based efficiency data for training (Kim *et al.* 2019, Wang *et al.* 2019, Xiang *et al.* 2021, Anthon *et al.* 2022). However, since the genomic context at surrogate targets is different from that of endogenous ones, variation in efficiency is observed between the two measurements, which nevertheless correlate well (Spearman’s *R* ≥ 0.7) (Kim *et al.* 2019, Wang *et al.* 2019, Xiang *et al.* 2021). Functional knockouts are an indirect measure of cleavage efficiency as not all indels resulting from cleavage events cause loss of function (e.g. knockout efficiency depends on the cleavage position and/or on the presence of specific domains (Shi *et al.* 2015, Doench *et al.* 2016)). Predicting functional knockout efficiency thus requires prediction of both target cleavage efficiency and of the effect of potential indels on the protein encoded by the targeted gene.

In the article “DeepCRISTL: deep transfer learning to predict CRISPR/Cas9 functional and endogenous on-target editing efficiency” published June 27th in *Bioinformatics*, 38, 2022, i161–i168 as part of the ISMB 2022 Proceedings, Elkayam and Orenstein applied transfer learning (TL) to improve Cas9 gRNA efficiency predictions at endogenous sites and for functional knockout tasks. After training an improved multi-task version of the DeepHF model, originally developed by Wang *et al.* (2019), on a large-scale dataset, Elkayam and Orenstein applied TL on each of 10 smaller datasets of endogenous cleavage and functional knockout data. The purpose was to improve the predictions of data originating from endogenous sites and functional knockouts. Different TL techniques were meticulously tested using the surrogate-based dataset of Wang et al. as the “source” dataset for pre-training and one of the 10 smaller endogenous or functional knockout datasets as the “target” for refining the TL model. By testing the refined models on held-out portions of the target datasets, the authors claim improved prediction ability compared to other available models, including our deep learning-based model, CRISPRon (Xiang *et al.* 2021).

Because a held-out portion of the data used for TL-based training was used for testing, we question if TL did in fact improve the prediction for endogenous cleavage or functional knockout or if, instead, it adjusted the predictions to fit a specific dataset. Hence, we were curious to see how a model refined on one target dataset “A” performs on another target dataset “B”, rather than on the held-out portion of A. It is our opinion that such comparison better reflects the generalization ability of model A and the actual performance that users will experience when relying on the predictions for their endogenous or knock-out experiments, as no re-fitting for the specific user’s methods and applications is possible without additional refinement on a dedicated target dataset.

The code for the DeepCRISTL models and the target data-sets used for TL were downloaded via https://github.com/OrensteinLab/DeepCRISTL (29 June 2022), while the trained models where at the time provided directly by the authors upon request (10 models were received while one model, Leenay, was not provided). The 10 datasets used as targets for these models were collected by Haeussler et al. (Haeussler *et al.* 2016) from publicly available data (Doench *et al.* 2014, Chari *et al.* 2015, Hart *et al.* 2015, Xu *et al.* 2015, Doench *et al.* 2016). For each dataset, Elkayam and Orenstein trained 10 models with different random initializations of the weights. The average output of these 10 models makes up the final DeepCRISTL model output for the given dataset. The performances presented by Elkayam and Orenstein were averaged over 5 repetitions in which a different held-out portion of the data was used as test set each time. However, only the models from one of these repetitions (“set_0”) were provided to us and therefore used here. Furthermore, for the xu2015TrainKbm7-dataset, model 5 was missing, hence we used model 0 twice.

**Figure 1.**
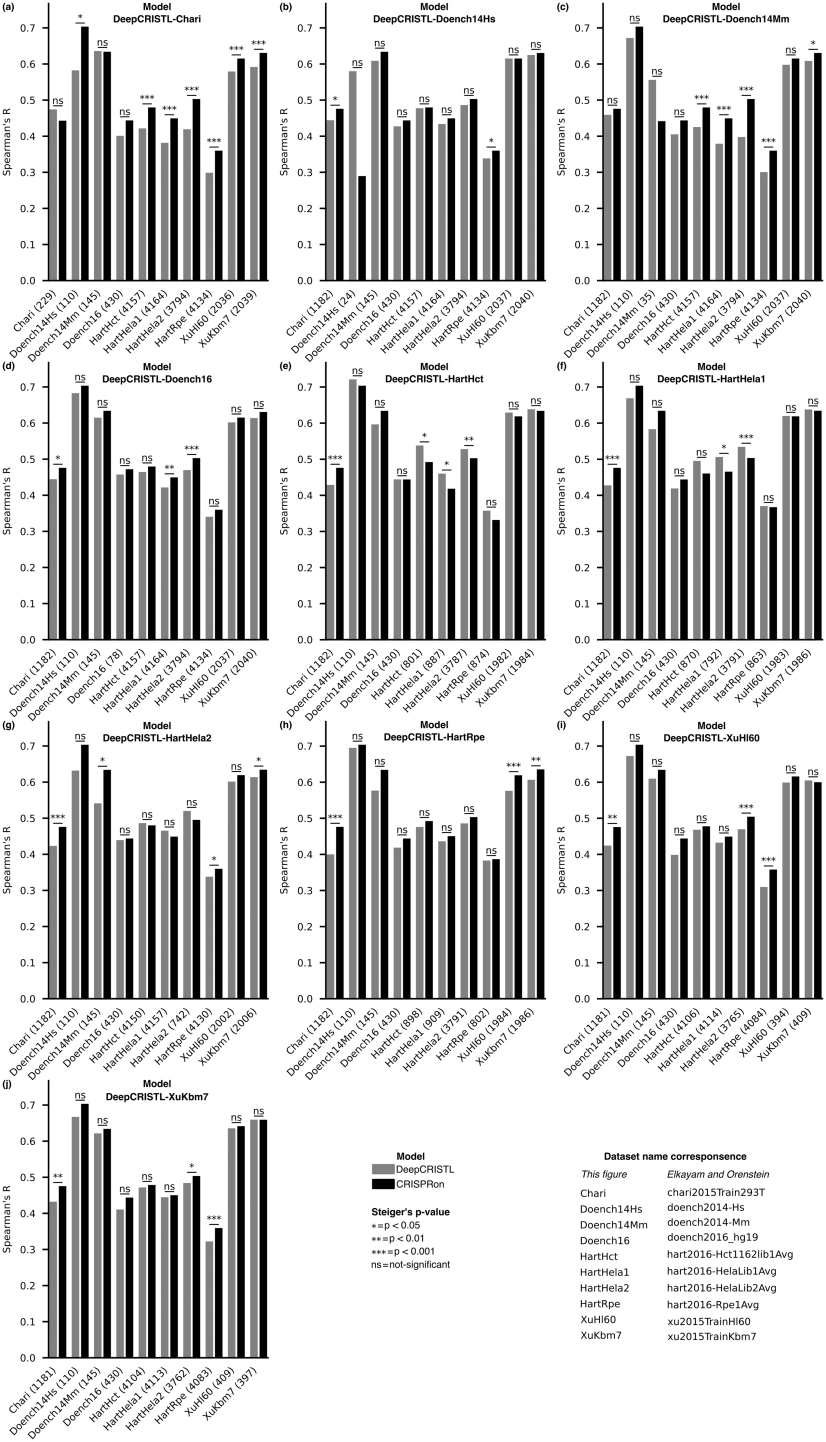
Comparison between DeepCRISTL and CRISPRon. (a–j) The Spearman’s correlation between the experimentally determined and the predicted efficiencies (DeepCRISTL, grey and CRISPRon, black) is displayed with vertical bars, one for each test dataset (*x*-axis). The name of the DeepCRISTL model used in each comparison is reported on top of the corresponding barplot. The number of gRNAs in each test dataset, after removing the overlap with the datasets used for training, is shown in parenthesis. Two-sided Steiger’s test *P*-values are reported for each comparison. The datasets and model names have been shortened for visualization purposes; the correspondence with the original the dataset and model names is displayed in the legend.

To compare the performances of CRISPRon and a specific DeepCRISTL model on each of the 10 held-out test datasets, we first removed gRNAs that were similar to gRNAs used in the training of either of the two models. Here, gRNAs are considered similar if their sequences (21 nt each, including 20 nt of gRNA and the first nucleotide in the NGG PAM) differ at less than 4 positions. For each of the similarity-reduced held-out datasets we compared the specific DeepCRISTL model with CRISPRon (Fig. 1), which gives 10 comparisons for each of the 10 models. Of the in total 100 comparisons, we notice that there is no significant difference in performance for most of them. However, there are notable differences. The two DeepCRISTL models trained on the data from HartHct and HartHela1 (Fig. 1e–f) surpass CRISPRon in five of six comparisons when tested on the datasets HartHela1, HartHela2 and HartHct, all of which are from the same provider. These 5 out of 100 comparisons are the only ones where a DeepCRISTL model significantly surpasses CRISPRon. In contrast, CRISPRon outperforms DeepCRISTL in 32 of the 100 comparisons. Notably, the two DeepCRISTL models trained on HartHela2 and HartRpe (Fig. 1g–h) do not outperform CRISPRon on any of the datasets from Hart *et al.*, while CRISPRon do surpass these two models on other datasets. It is also worth noticing that CRISPRon outperforms the DeepCRISTL model trained on the dataset of Chari *et al.* on seven of the test datasets (Fig. 1a).

Removing the overlap between training and test data substantially reduces the size of the test datasets, and this impacts the evaluation of the differences between models. For instance, the predictions of the DeepCRISTL model trained on the Doench14Hs data have higher correlation (Spearman’s *R* = 0.58) compared to CRISPRon (*R* = 0.29) to the efficiencies in the Doench14Hs test set which, however, only contains 24 gRNAs (Fig. 1b). Thus, the difference between these two correlations (*R* = 0.67 between the model’s predictions) is not significant by our significance cutoff of 0.05 (*P *=* *.054). Despite the loss of data, filtering the test set for overlap with the training sequences remains a necessary procedure for a realistic and fair performance evaluation.

Because of the overlaps between the different datasets, it was not possible to make a single comparison with sufficient test data that included all the DeepCRISTL models to inspect their prediction performances. However, because some datasets do not overlap, or overlap minimally, with others, we can still observe part of the variability between the DeepCRISTL models. For instance, the prediction performance of the DeepCRISTL models on the dataset of Chari *et al.* varies between 0.40 and 0.46. Similarly, on the Doench16 dataset, DeepCRISTL models’ performance varies from 0.40 to 0.44. The variation in performance implies differences in the predictions provided by the DeepCRISTL models and suggests that the models are not interchangeable to predict, e.g. protein loss of function. Part of this variability could be overcome by integrating together multiple datasets; however, this task is challenging as different techniques were applied in each study, hence the efficiency values in the datasets are not directly comparable.

In our opinion, it is essential to state why the data selected for model testing can be deemed independent from the training data. In this respect we agree with the requirements covered by the bioinformatics instructions for authors and more recently by the DOME (Data, Optimization, Model, Evaluation) recommendations for supervised machine learning validation in biology (Walsh *et al.* 2021). The application of TL further complicates the assessment of train-test data independence as there may be factors, such as lab techniques or evaluation methods, which make a dataset “unique”, thus allowing dataset-specific fitting during TL which may not generalize to otherwise similar datasets. Most of these factors are not used as model features nor considered during train-test splitting because it would be unfeasible to systematically test the impact of their full range of options (e.g. the cell line used, the timing of each activity in the process). Therefore, to substantiate how the performance improvement given by TL generalizes, it is necessary to use external test data that does not come from the same data source as the one used for refinement in the training.

We acknowledge the potential of applying TL to improve gRNA efficiency predictions at endogenous sites and for functional knockout tasks. However, our analysis emphasizes the importance of using external test datasets for performance evaluation. We find that (i) DeepCRISTL only gains performance over CRISPRon in 5 of 100 comparisons and that all of these are on held-out data from the same provider of the dataset used for training, (ii) CRISPRon outperforms the DeepCRISTL models in 32 comparisons, all involving data unrelated to the CRISPRon training set, and (iii) there are no differences in 63 comparisons. We conclude that TL did not lead to an improved performance on external datasets and that CRISPRon is the overall best performing model.

Conflict of interest: none declared.
